# Free triiodothyronine (FT3)-to-free thyroxine (FT4) ratio identified as a risk factor for gestational diabetes in euthyroid pregnant women: insights from a Chinese population cohort study

**DOI:** 10.3389/fendo.2023.1281285

**Published:** 2023-11-20

**Authors:** Xin Zhao, Jianbin Sun, Ning Yuan, Xiaomei Zhang

**Affiliations:** Department of Endocrinology, Peking University International Hospital, Beijing, China

**Keywords:** thyroid hormone, gestational diabetes mellitus, oral glucose tolerance test, glycosylated hemoglobin, pregnancy

## Abstract

**Background:**

To explore the association between thyroid hormones and gestational diabetes mellitus in euthyroid pregnant women, with the aim of preventing the occurrence of gestational diabetes mellitus.

**Methods:**

In this prospective study, a total of 1222 euthyroid pregnant women in their first trimester were recruited at Peking University International Hospital between December 2017 and March 2019. These participants underwent an oral glucose tolerance test during the 24-28 weeks of gestation.

**Results:**

During early pregnancy, the gestational diabetes mellitus group displayed lower levels of free thyroxine when compared to the non-gestational diabetes mellitus group. Additionally, the ratio of free triiodothyronine to free thyroxine in the gestational diabetes mellitus group during early pregnancy was significantly higher (p<0.05). The ratio of free triiodothyronine to free thyroxine during early pregnancy showed a positive correlation with blood glucose levels at 0, 60, and 120 min both before and after glucose loading (all p<0.05). During early pregnancy, there was a negative relationship between free thyroxine levels and fasting blood glucose. The free triiodothyronine levels were positively correlated to blood glucose levels at 120 min following glucose loading (all p<0.05).

**Conclusion:**

The ratio of free triiodothyronine-to-free thyroxine is an independent risk factor for gestational diabetes mellitus and has the potential to be a predictor for gestational diabetes mellitus in euthyroid pregnant women.

## 1 Introduction

As the economic level has risen, there has been a notable increase in the risk of various endocrine disorders during pregnancy (1), including gestational diabetes mellitus (GDM), thyroid disease (TD), hyperlipidemia, and so on. GDM is a frequent complication of pregnancy that can have negative impacts on the well-being of both mothers and their children (2). Thus, early detection and treatment of GDM are advisable (3) and an exploration of risk factors associated with the development of GDM would provide valuable clinical insights and benefits.

The intricate physiological transformations that occur during pregnancy also influence the metabolic alterations in thyroid function. Recent studies, both domestic and international, have explored how abnormalities in thyroid hormone (TH) levels are associated with the occurrence of GDM through different mechanisms (4, 5). Nonetheless, there is still a dearth of evidence-based research regarding the association between GDM and thyroid hormone (TH) during the first trimester of pregnancy, and the underlying mechanisms of this association remain unclear.

A recent study found that initiating treatment for gestational diabetes before the 20th week of pregnancy resulted in a slightly reduced occurrence of a combination of negative neonatal outcomes compared to no early treatment (6). In clinical practice, for women identified as being at a high risk for GDM, early implementation of lifestyle interventions is essential to minimize the incidence of GDM.The objective of this study is to study the association between TH in early pregnancy and the development of GDM and to identify predictive factors for the occurrence of GDM.

## 2 Materials and methods

### 2.1 Ethics statement

The study was approved by the Ethics Bioethics Committee of Peking University International Hospital. All protocols followed the ethical guidelines of the institution and national committee and complied with the 1964 Declaration of Helsinki and subsequent amendments. All participants provided written informed consent.

### 2.2 General information

The age, parity, family history, and personal history of GDM, family history of TD, and the gestational week of pregnant women were recorded at the time of enrollment, data on blood pressure (both systolic blood pressure and diastolic blood pressure), as well as measurements of height and weight, were obtained, and body mass index was calculated and recorded. BMI was calculated using the formula BMI (kg/m^2^) = weight(kg)/body height^2^(m^2^). The pregnant women participating in this study had their fasting blood glucose levels and TH including antibodies measured before becoming pregnant.

Sample size calculation: The sample size calculation formula for survey research is used to determine the sample size: n=U α ^2^ * π (1- π)/δ ^2^. The π is the overall rate of GDM and π=0.20, δ is for an error of 2%, and U α= 1.96. Therefore, 1,537 subjects will be included in this study.

Inclusion criteria for the study were as follows: (1) Age of 18 years or older. (2) Willing to undergo an oral glucose tolerance test (OGTT) between the 24th and 28th weeks of gestation. (3) Planned to receive prenatal check-ups and deliver their baby at the hospital. (4) Consented to participate in the relevant questionnaire survey and agreed to the collection of blood samples after being informed about the content of the survey.

**Figure 1:**
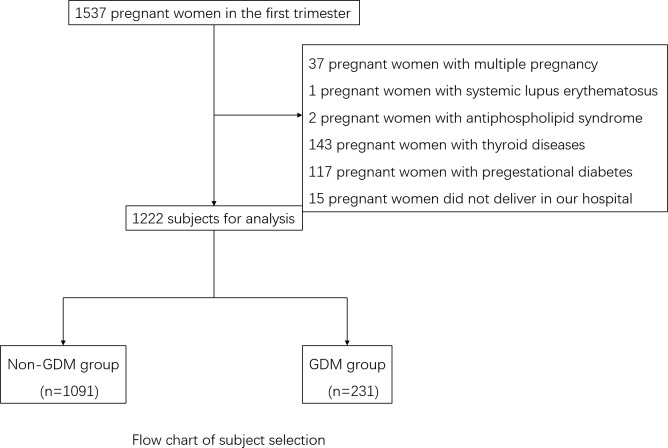
Flow chart of subject selection.

The exclusion criteria for the study were as follows: (1) Pre-existing diagnoses of cardiovascular, cerebrovascular, hematological, liver, renal, or respiratory diseases, or pre-pregnancy diabetes mellitus, or pre-thyroid diseases including positive antibodies. (2) Carrying multiple pregnancies. (3) Lack of essential baseline data. Finally, 1,222 subjects with complete data were recruited in this study (**Figure 1**).

### 2.2 Biochemical index detection

All the subjects had fasting 5 ml of venous blood collected in the morning during the 7-12 weeks of their gestation period. The detection indexes included glycosylated hemoglobin (HbA1c), fasting blood glucose (FBG), total cholesterol (TC), triglycerides (TG), high-density lipoprotein cholesterol (HDL-C), low-density lipoprotein cholesterol (LDL-C), uric acid (UA), and serum creatinine (SCr). Additionally, the TH levels were measured, including total thyroxine (TT4), total triiodothyronine (TT3), free thyroxine (FT4), free triiodothyronine (FT3), thyroid-stimulating hormone (TSH), thyroid peroxidase antibodies (TPOAb), and thyroglobulin antibodies (TgAb), and FT3/FT4 ratio was calculated. The biochemical indices were analyzed in the laboratory of Peking University International Hospital Center. HbA1c levels were determined using high-performance liquid chromatography (HPLC) and a Dongcao G8 analyzer.

### 2.3 Diagnosis of GDM

The pregnant women were screened for GDM through a 75g OGTT at 24–28 weeks of gestation. To perform this test, the pregnant women were admitted to the hospital in the morning after fasting for 8–12 h. They were provided with 75g of glucose powder, which was dissolved in 250ml–300ml of warm boiled water, and they had to consume it quickly within 5 min. Blood glucose levels were measured at three specific time points during the oral glucose tolerance test: before taking the glucose solution (GLU0min), at 1 hour after taking the glucose solution (GLU60min), and at 2 h after taking the glucose solution (GLU120min).

The diagnostic criteria for GDM in this study were based on the IADPSG (International Association of the Diabetes and Pregnancy Study Groups) guidelines (7). According to these criteria, the blood glucose values at different time points during the OGTT should be as follows: GLU0min should be lower than 5.1 mmol/L, GLU60min should be lower than 10.0 mmol/L, and GLU120min should be lower than 8.5 mmol/L. If any of the blood glucose values reach or exceed these specified criteria, a diagnosis of GDM is made.

The weight of the pregnant women at 24–28 weeks of gestation was documented, and their weight gain during this period was calculated and recorded.

### 2.4 Statistical analysis

All data were analyzed using SPSS 22.0. Data were tested for normality. Normally distributed data were expressed as means ± standard deviation (x ± s) and compared using t-tests, while non-normally distributed data were expressed as medians (P25, P75) and compared using rank sum tests. The counting data were expressed as rates, and comparisons between the two groups were made using χ2 tests. Spearman correlations were used to assess associations between variables, while univariate and multivariate analyses were conducted using unconditional logistic regression models. These models were used to calculate the odds ratio (OR) and its corresponding 95% confidence interval (95%CI). Receiver operating characteristic (ROC) curves were plotted, and the areas under the curve (AUC) were calculated. All statistical tests were two-sided, and p<0.05 was considered statistically significant.

## 3 Results

### 3.1 Comparison of general conditions and biochemical indexes between the two groups in the first trimester of pregnancy and OGTT results

Out of the 1,222 patients, 231 were diagnosed with GDM during the second trimester, resulting in an incidence rate of 18.90%. All the patients tested negative for TPOAb and TGAb. The levels of HbA1c ranged from 4.0% to 6.4% in the non-GDM group and 4.5% to 9.5% in the GDM group. In comparison to the non-GDM group, there was a notable increase in the proportion of individuals with a personal history and family history of GDM in the GDM group (χ2 = 10.21 and χ2 = 9.87, all p<0.05). When compared to the non-GDM group, there was a significant increase in the proportion of multipara in the GDM group (χ2 = 9.94, p<0.05). Women with GDM tended to have higher BMI than those without and also showed higher levels of both HbA1c and FBG in the first trimester of pregnancy (all p<0.05). The levels of TG TC, LDL-C, and UA were also higher in the GDM group (all p<0.05). Women with GDM also showed lower levels of FT4 and a significantly higher FT3/FT4 ratio than those without GDM (all p<0.05). The levels of TT4, TT3, FT3, and TSH did not differ significantly between the two groups during early pregnancy (all p>0.05) (**Table 1**).

**Table 1:** Comparison of general conditions and biochemical indexes between the two groups in the first pregnancy and OGTT results.

Index	non-GDM group	GDM group	t(X2)	p
	(n=991)	(n=231)		
Age (years)	30.94 ± 3.64	30.77 ± 3.86	0.63	0.52
BMI (kg/m^2^)	21.65 ± 3.01	23.31 ± 3.16	-7.62	<0.05
Personal history of GDM	10 (1.01%)	43(18.61%)	10.21	<0.05
Family history of GDM	5 (0.50%)	21(9.10%)	9.87	<0.05
Family history of TD	37(3.73%)	11(4.76%)	3.21	0.12
Parity				
0	588(59.33%)	111(48.05%)	9.94	<0.05
≥1	403(40.67%)	120(51.95%)		
SBP (mmHg)	110.06 ± 10.64	109.71 ± 10.26	0.45	0.65
DBP (mmHg)	66.48 ± 8.91	64.77 ± 9.08	2.62	<0.05
TC (mmol/L)	3.93 ± 0.69	4.06 ± 0.67	-2.69	<0.05
TG (mmol/L)	0.95 ± 0.58	1.13 ± 0.47	-4.46	<0.05
LDL-C(mmol/L)	2.03 ± 0.55	2.12 ± 0.54	-2.28	<0.05
HDL-C(mmol/L)	1.41 ± 0.28	1.42 ± 0.29	-0.51	0.61
UA (umol/L)	211.92 ± 46.54	227.80 ± 47.95	-4.67	<0.05
sCr (umol/L)	49.67 ± 7.11	48.98 ± 6.78	1.33	0.18
HbA1c (%)	5.08 ± 0.26	5.29 ± 0.30	-5.62	<0.05
FBG (mmol/L)	4.87 ± 0.40	5.04 ± 0.41	-6.19	<0.05
gestational weight gain(kg)	9.34 ± 1.23	12.09 ± 2.32	-5.43	<0.05
FT4 (pmol/l)	16.84 ± 1.92	16.40 ± 1.95	3.13	<0.05
FT3 (pmol/l)	4.62 ± 0.50	4.67 ± 0.50	-1.40	0.16
TT4 (nmol/l)	121.01 ± 22.24	118.88 ± 20.70	1.31	0.19
TT3 (nmol/l)	1.76 ± 1.19	1.83 ± 1.09	-0.93	0.35
TSH (uIU/ml)	2.03 ± 0.35	2.06 ± 0.36	-1.16	0.25
FT3/FT4	0.27 ± 0.04	0.29 ± 0.04	-3.92	<0.05

BMI is for body mass index, SBP is systolic blood pressure, DBP is for diastolic blood pressure, FBG is for fasting blood glucose, HbA1c is for glycosylated hemoglobin, sCr is for serum creatinine, UA is for uric acid, TC is for total cholesterol, TG is for triglycerides, LDL-C is for low-density lipoprotein cholesterol, HDL-C is for high-density lipoprotein cholesterol, TT4 is for total thyroxine,TT3 is for total triiodothyronine, FT4 is for free thyroxine, FT3 is for free triiodothyronine, TSH is for thyroid-stimulating hormone, GLU0min is for fasting blood glucose before OGTT, GLU60min is for blood glucose 60 min after OGTT, GLU120min is for blood glucose 120 min after OGTT.

### 3.2 Association between TH and blood glucose before and after glucose loading

Positive correlations were found between BMI, TG, TC, LDL-C, UA, and blood glucose levels before and after glucose loading (all p<0.05). There were positive correlations between weight gain and blood glucose levels before and after glucose loading (all p<0.05). There were positive correlations between FT3/FT4 ratio in the first trimester of pregnancy and blood glucose levels before and after glucose loading (all p<0.05). The FT4 levels in the first trimester were negatively correlated with GLU0min and FT3 levels were positively correlated with GLU120min (all p<0.05). There were no significant correlations between TT3, TT4, TSH, and glucose levels before and after glucose loading (all p>0.05) (**Tables 2**, **3**).

**Table 2:** Correlation Analysis between biochemical indexes and blood glucose before and after glucose loading.

Index	GLU0min		GLU60min		GLU120min	
	r	p	r	p	r	p
BMI (kg/m^2^)	0.25	<0.05	0.18	<0.05	0.26	<0.05
SBP (mmHg)	-0.06	0.06	-0.07	<0.05	-0.03	0.38
DBP (mmHg)	-0.07	<0.05	-0.10	<0.05	-0.07	<0.05
TC (mmol/L)	0.08	<0.05	0.12	<0.05	0.13	<0.05
TG (mmol/L)	0.12	<0.05	0.15	<0.05	0.15	<0.05
LDL-C(mmol/L)	0.11	<0.05	0.12	<0.05	0.12	<0.05
HDL-C(mmol/L)	-0.10	<0.05	-0.01	0.84	-0.04	0.14
UA (umol/L)	0.16	<0.05	0.14	<0.05	0.16	<0.05
sCr(umol/L)	-0.02	0.54	-0.01	0.70	-0.03	0.30
gestational weight gain(kg)	0.09	<0.05	0.04	0.09	0.11	<0.05

BMI is for body mass index, SBP is systolic blood pressure, DBP is for diastolic blood pressure, FBG is for fasting blood glucose, HbA1c is for glycosylated hemoglobin, sCr is for serum creatinine, UA is for uric acid, TC is for total cholesterol, TG is for triglycerides, LDL-C is for low-density lipoprotein cholesterol, HDL-C is for high-density lipoprotein cholesterol.

**Table 3:** Correlation Analysis between thyroid hormone and blood glucose before and after glucose loading.

Index	GLU0min		GLU60min		GLU120min	
	r	p	r	p	r	p
FT4 (pmol/l)	-0.06	<0.05	-0.05	0.06	-0.04	0.20
FT3 (pmol/l)	0.03	0.34	0.05	0.09	0.06	<0.05
TT4 (nmol/l)	-0.01	0.67	-0.03	0.31	-0.03	0.29
TT3 (nmol/l)	0.02	0.46	0.03	0.39	0.02	0.28
TSH (uIU/ml)	0.06	0.06	0.06	0.06	0.05	0.05
FT3/FT4	0.07	<0.05	0.08	<0.05	0.09	<0.05

TT4 is for total thyroxine, TT3 is for total triiodothyronine, FT4 is for free thyroxine, FT3 is for free triiodothyronine, TSH is for thyroid-stimulating hormone, GLU0min is for fasting blood glucose before OGTT, GLU60min is for blood glucose 60 min after OGTT, GLU120min is for blood glucose 120 min after OGTT.

### 3.3 Logistic regression analyses of TH and GDM

Multivariate logistic regression was conducted using GDM as the dependent variable and variables that were shown to be significant in the univariate analysis as independent variables. After adjustment for age, BMI, parity, blood lipid, blood pressure, UA, and sCr, the FT3/FT4 ratio was an independent risk factor for GDM (**Table 4**).

**Table 4:** Logistic regression between thyroid function in first trimester and GDM.

Index	Crude OR	95%CI	p	Adjust OR	95%CI	p
FT4(pmol/L)						
Low:12.01-16.70	1			1		
Medium:16.71-18.86	0.78	0.55-1.09	0.59	0.84	0.58,1.20	0.33
High:18.87-22.00	0.67	0.47-0.95	<0.05	0.75	0.52-1.09	0.13
FT3(pmol/L)						
Low:3.20-4.62	1			1		
Medium:4.62-4.81	1.27	0.86-1.85	0.23	1.22	0.82-1.82	0.32
High:4.81-6.39	1.41	0.98-2.03	0.06	1.32	0.90-1.94	0.15
FT3/FT4						
Low:0.17-0.28	1			1		
Medium:0.28-0.32	1.58	1.08-2.30	<0.05	1.45	1.01-2.01	<0.05
High:0.32-0.41	1.95	1.35-2.80	<0.05	1.67	1.14-2.46	<0.05

FT4 is for free thyroxine, FT3 is for free triiodothyronine.

### 3.4 Single variable predicting model of GDM

The model for predicting the risk of GDM using individual variables including FT3, FT4 and FT3/FT4 showed that the AUCs were ranked FT3/FT4 (0.59) > FT4 (0.57) > FT3 (0.51). The cut-off points of FT4, FT3, and FT3/FT4 were 15.55 pmol/L, 119.7 pmol/L, and 0.27, respectively (**Table 5**).

**Table 5:** Univariate predictive model of GDM.

Index	AUC (95%CI)	Specificity	Sensitivity	Cut-off
FT4	0.57 (0.53, 0.61)	0.74	0.35	15.55
FT3	0.51 (0.47, 0.56)	0.54	0.54	4.65
FT3/FT4	0.59 (0.55, 0.63)	0.45	0.68	0.27

TT4 is for total thyroxine, FT4 is for free thyroxine, FT3 is for free triiodothyronine.

**Figure 2:**
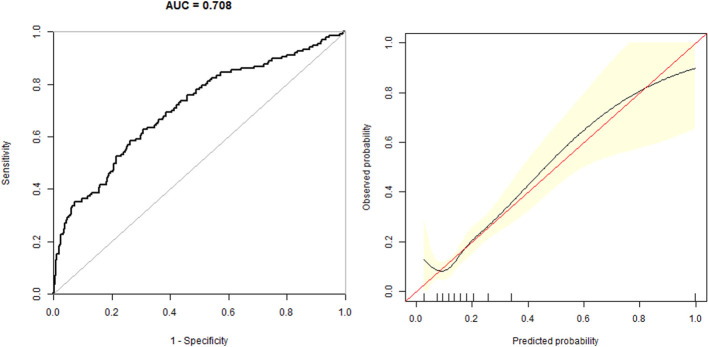
The overall predictive accuracy of multivariate predictive model for the risk of GDM. The AUC is 0.708 (95% CI 0.66, 0.76), a specificity of 73.83%, a sensitivity of 58.39% and an accuracy of 70.87%.

In the multivariate predictive model, GDM was used as the dependent variable, and age, BMI, parity, blood lipid, blood pressure, UA, and FT3/FT4 were used as independent variables. The regression equation is - 11.61649 + 6.46352 * FT3/FT4 + 0.00026 * Age + 0.12568 * BMI + 0.10806 * TC + 0.07897 * TG-0.006666 * SBP + 0.44134 * Parity + 1.07293 * HbA1c. The model had an AUC of 0.708 (95% CI 0.66, 0.76), a specificity of 73.83%, a sensitivity of 58.39%, and an accuracy of 70.87% (**Figure 2**).

## 4 Discussion

GDM can result in significant perinatal complications, including macrosomia, shoulder dystocia, cesarean section, and neonatal hypoglycemia, Additionally, GDM can have long-term effects on the mother’s risk of developing type 2 diabetes mellitus (T2DM) and can contribute to obesity in the child. During pregnancy, the thyroid gland enlarges to meet the increased hormonal demands of pregnancy, growing by approximately 40%. This expansion is accompanied by changes in the secretion of FT3 and FT4 levels and an overall increase in metabolic activity (8).

TH plays a crucial role in regulating balanced glucose metabolism. Its involvement in insulin signal transduction and the maintenance of glucose homeostasis is considered a potential factor in human pathophysiology. Both a deficiency or an excess of TH can disrupt the normal regulation of glucose in the body. T3 is the main bioactive hormone responsible for glucose-related metabolic activities. Elevated TSH levels can cause harm to the pancreatic islets and possibly hinder the function of beta cells, resulting in insulin resistance and elevated blood glucose levels (8). Studies in rats with subclinical hypothyroidism (SCH) have shown a reduction in glucose production in the liver, along with a decreased rate of glucose utilization in skeletal muscles and adipose tissues (9, 10).

Our results demonstrated a significant association between the FT3/FT4 ratio and blood glucose levels in the second trimester of pregnancy. It was found that the FT3/FT4 ratio was the only parameter that showed a significant positive correlation with blood glucose levels after glucose loading during the second trimester. T4 is usually considered as a pre-hormone. As the precursor to the biologically active form, T3, the FT3/FT4 ratio is utilized to assess deiodinase activity. In a cross-sectional study, it was observed that elevated deiodinase activities in women with normal TH levels were associated with higher BMI and increased deiodinase activity was significantly correlated with higher blood glucose levels. It is theorized that the increased deiodinase activity triggered by BMI may elevate the risk of GDM by amplifying the effects of T3 (11). A recent study has reported similar findings, indicating that a higher FT3-to-FT4 ratio during the later stages of pregnancy was linked to an increased risk of GDM, adverse pregnancy outcomes, and an adverse metabolic profile in the early postpartum period (12). Nonetheless, it is worth noting that the study had a relatively small sample size, and it was unable to demonstrate the predictive value of FT3/FT4 for the occurrence of GDM. Another study, however, did establish associations between FT3/FT4 ratio in the first trimester and GLU0min and concluded that FT3/FT4 was an independent risk factor for the development of GDM. This finding aligns with the outcomes of our study (13, 14). However, the outcomes of these studies also indicated significant associations between FT3 and TSH levels and GDM. It is important to note that the subjects in these studies consisted of pregnant women with SCH or T4 levels, which might introduce confounding variables and affect the analysis of the relationship between TH and GDM in euthyroid women. Another study with large number subjects has shown that lower concentration of serum FT4 or higher FT3/FT4 ratio in early pregnancy was associated with an increased risk of GDM (OR = 1.43; 95% CI 1.06, 1.93, p= 0.01) after adjusting for potential confounders. However, the study failed to find the cut-off of the FT3/FT4 ratio for predicting GDM (15). Our study focused on euthyroid women during the first trimester of pregnancy, intentionally excluding women with TD or those taking medication that could potentially confound the analysis of the relationship between TH and GDM. In our research, we employed ROC analysis to evaluate the predictive value of FT3, FT4, and the FT3/FT4 ratio for the occurrence of GDM. The AUCs were ranked from smallest to largest as follows: FT3/FT4 (0.59) > FT4 (0.57) > FT3 (0.51). Additionally, it was determined that the cut-off points for FT4, FT3, and FT3/FT4 in predicting GDM were 15.55 pmol/L, 119.7 pmol/L, and 0.27, respectively.

The interaction between TH and blood glucose may be influenced by several factors. (1) TH has the capacity to regulate the expression of glucose transporter 2 (GLUT2) (11). Intrahepatic gluconeogenesis is a process that can lead to the swift transport of glucose across the cytoplasmic membrane of the liver. This glucose efflux, which occurs at the hepatic cytoplasmic membrane, is facilitated by a protein called GLUT2. The pathway involving gluconeogenesis, kinesin, and glucose transporter interactions may have secondary effects on hepatocytes and could result in reduced sensitivity to insulin in the liver (16). Some studies have shown that T3 can induce the expression of glucose transporter 4 (GLUT4) and GLUT4 is known to potentially enhance insulin sensitivity (17), therefore, T3 might have the capacity to induce insulin sensitivity. (2) It has been confirmed that abnormal TH levels may have adverse effects on mitochondrial function (18). T3 has the ability to directly bind to specific T3 binding sites in mitochondria. Additionally, it can exert its influence on the cell nucleus, indirectly impacting the transcription of genes associated with the regulation of cellular metabolism and mitochondrial function (19). Mitochondria play a crucial role in glucose metabolism within pancreatic cells. Any defects in mitochondrial function can make individuals more susceptible to cellular dysfunction, which, in turn, may increase the risk of developing T2DM.

Our study observed slightly higher TT3 and FT3 levels in patients with GDM than those without GDM, and the FT3 level was positively associated with the GLU120min level after glucose loading (r=0.06, p<0.05). However, after adjusting for BMI, blood pressure, and other variables in logistic regression, the association between FT3 and GDM risk was not sustained.

In a retrospective analysis involving a total of 27,513 pregnant women, which included 3,697 cases in the GDM group, the relationship between various TH levels and GDM was examined. The findings revealed that pregnant women with GDM had lower FT4 levels in comparison to those without GDM (p<0.01). A lower FT4 level during the first trimester of pregnancy was found to be linked with the development of GDM (p<0.01) (20). Many research studies have verified the association between reduced FT4 levels during the second and third trimesters of pregnancy and an elevated risk of GDM. Nevertheless, the exact cause-and-effect relationship between these two factors remains unclear. A meta-analysis showed that isolated maternal hypothyroxinaemia (IMH) was associated with increased GDM, preterm premature rupture of membranes, preterm birth, fetal distress, and macrosomia outcomes in IMH compared to euthyroid women, and the relative risks were 1.42, 1.50, 1.33, 1.75 and 1.62, respectively. IMH was not associated with placenta previa, gestational hypertension, pre-eclampsia, intrauterine growth restriction, and off-spring outcomes like birth weight, low birth weight infants, fetal macrosomia, neonatal intensive care, neonatal death, or fetal head circumference (21). However, certain studies have indicated that, even after accounting for confounding variables, there is no statistically significant correlation between FT4 and GDM (6, 22). The majority of previous research has primarily focused on pregnant women with SCH or low T4 levels, but there has been a scarcity of research findings concerning pregnant women with normal TH. In our study, we identified a negative correlation between FT4 and blood glucose levels following OGTT in pregnant euthyroid women.

In addition, it has been reported that TSH binding to adipocyte receptors stimulates the secretion of IL-6 from the cells, leading to proliferation, differentiation, and leptin production in both preadipocytes and adipocytes (23). These findings indicate that adipocytes may play a role in connecting insulin resistance with TSH. A recent study identified a positive association between TSH levels and the homeostasis model assessment of insulin resistance (HOMA-IR) in both individuals with diabetes and those without diabetes. This observation suggests an independent and direct correlation between insulin resistance and TSH levels (24). A population-based Chinese study also showed an independent correlation between TSH during the first trimester of pregnancy and GDM. This association was particularly significant among women with higher BMI values prior to pregnancy (25). In our own study, we did not find this association, which could be attributed to the fact that the study participants had normal TH levels, and their TSH levels fell within the normal range. This did not allow us to determine whether the risk of GDM increases when TSH levels exceed the normal range.

Some studies have shown that thyroid disease and GDM could share some common risk factors, such as age, BMI, vitamin D deficiency, selenium level and so on (26). Additionally, our study identified parity, BMI, blood pressure, blood lipid, UA, and HbA1c levels as independent risk factors for GDM, confirming the findings of earlier studies (27, 28). In order to avoid the biases, the multivariate logistic regression was conducted using GDM as the dependent variable after adjustment for age, BMI, parity, blood lipid, blood pressure, UA, and sCr. In our investigation, we established a predictive model with GDM as the dependent variable and age, parity, blood pressure, BMI, blood lipid, HbA1c, UA, and FT4/FT3 as independent variables. The AUC of the prediction model was 0.708 (95% CI 0.66,0.76), the specificity was 73.83%, the sensitivity was 58.39%, and the accuracy was 70.87%, which indicates that the model possesses a certain degree of predictive value for the early diagnosis of GDM.

The study has several limitations. Firstly, the subjects were enrolled from a single hospital and the sample size was limited. In future research, it would be beneficial to involve multiple centers and a larger sample of subjects to enhance the generalizability of the findings. Secondly, our study solely incorporated TH data from the first trimester of pregnancy, and we did not perform a dynamic assessment of TH throughout the entire pregnancy. In future research, it would be valuable to include TH measurements from various stages of pregnancy to provide a more comprehensive understanding of the relationship between TH and GDM. Furthermore, in future studies, we plan to conduct a more extensive investigation into the correlation between TH and insulin sensitivity as well as insulin resistance. This will help clarify the specific relationship between TH and glucose metabolism. Finally, the correlation analysis between TH and blood glucose revealed statistically significant but not particularly high correlation values (r values). To better elucidate the causal relationship between TH and blood glucose, we further conducted a logistic regression analysis. The results of our logistic regression analysis have indicated a causal relationship between TH and diabetes. We believe that while the r values may not be very high, their statistical significance still suggests a trend in the correlation between these two variables. In future research, as the sample size continues to grow, it may become more likely to observe a stronger and more significant linear relationship between the variables.

## 5 Conclusions

This study delved into the association between the FT3/FT4 ratio and GDM in pregnant women with normal thyroid function. It is worth noting that the study featured a substantial sample size, effectively excluding the influence of abnormal TH on the research outcomes. Furthermore, we constructed a prediction model for GDM based on the FT3/FT4 ratio, and an ROC curve was generated to evaluate its performance. The study also identified a specific cut-off value for predicting GDM. The FT3/FT4 ratio is an independent risk factor for GDM in the first trimester of pregnancy. When compared with the individual components, FT4 and FT3, the FT3/FT4 ratio exhibits greater predictive value for the development of GDM.

## Data availability statement

The raw data supporting the conclusions of this article will be made available by the authors, without undue reservation.

## Ethics statement

The studies involving humans were approved by bioethics committee of Peking University International Hospital. The studies were conducted in accordance with the local legislation and institutional requirements. The participants provided their written informed consent to participate in this study.

## Author contributions

XZ: Conceptualization, Formal Analysis, Methodology, Software, Writing – original draft. JS: Conceptualization, Formal Analysis, Methodology, Software, Writing – review & editing. NY: Data curation, Writing – review & editing. XMZ: Funding acquisition, Project administration, Supervision, Writing – review & editing.
